# Andexanet Alfa versus Four-Factor Prothrombin Complex Concentrate for the Reversal of Factor Xa (FXa) Inhibitor-Associated Intracranial Hemorrhage: A Systematic Review of Retrospective Studies

**DOI:** 10.3390/jcm13113077

**Published:** 2024-05-24

**Authors:** Luan Oliveira Ferreira, Ricardo Andres León Oldemburg, João Monteiro Leitão Filho, Rodrigo Arcoverde Cerveira, Victoria Winkler Vasconcelos, Giovana Escribano da Costa, Roseny dos Reis Rodrigues, Dielly Catrina Favacho Lopes

**Affiliations:** 1 Department of Anesthesiology, João de Barros Barreto University Hospital, Belém 66073-000, Brazil; andresper@hotmail.com (R.A.L.O.); jfilho_monteiro@icloud.com (J.M.L.F.); victoria.winkler16@gmail.com (V.W.V.); giovanaescribano@gmail.com (G.E.d.C.); 2 Division of Immunology & Allergy, Department of Medicine Solna, Karolinska Institutet, Karolinska University Hospital, SE-171 76 Stockholm, Sweden; rodrigo.arcoverdi@gmail.com; 3 Center for Molecular Medicine, Karolinska Institutet, Karolinska University Hospital, SE-171 76 Stockholm, Sweden; 4 Department of Anesthesiology, São Paulo University, São Paulo 05403-010, Brazil; ny_rodrigues@yahoo.com.br; 5 Intensive Care Department, Albert Einstein Hospital, São Paulo 05652-900, Brazil; 6 Laboratory of Experimental Neuropathology, Federal University of Pará, Belém 66073-000, Brazil; dclopes@ufpa.br

**Keywords:** Andexanet alfa, four-factor prothrombin complex concentrate, intracranial hemorrhage, factor Xa inhibitors, mortality

## Abstract

**Background/Objectives**: There are limited data on the risks and benefits of using Andexanet alfa (AA) compared with four-factor prothrombin complex concentrate (4F-PCC) for the reversal of factor Xa inhibitor-associated intracranial hemorrhage (ICH). Our aim was to describe a compilation of the information available in the literature to date. **Methods**: PubMed, Embase, Web of Science (Clarivate Analytics) and the Cochrane Central Register of Controlled Trials were searched until December 2023. Following the “Preferred Reporting Items for Systematic Reviews and Meta-Analyses (PRISMA)” guidelines, our systematic literature review included studies that were retrospective in design and evaluated both drugs to control bleeding and complications (death and thromboembolic events). Two researchers re-examined the studies for relevance, extracted the data and assessed the risk of bias. No meta-analyses were performed for the results. **Results**: In this limited patient sample, we found no differences between published articles in terms of neuroimaging stability or thrombotic events. However, some studies show significant differences in mortality, suggesting that one of the AAs may be superior to 4F-PCC. **Conclusions**: Our qualitative analysis shows that AA has a better efficacy profile compared with 4F-PCC. However, further studies monitoring these patients and a multicenter collaborative network dedicated to this topic are needed.

## 1. Introduction

Since their approval by the Food and Drug Administration (FDA) and the European Medicines Agency (EMA) in 2010, direct oral anticoagulants (DOACs) and, in particular, the FXa inhibitors apixaban and rivaroxaban have been increasingly used to treat or prevent various thrombotic diseases [1,2,3,4].

Although the use of DOACs is associated with a lower risk of intracranial hemorrhage compared to vitamin K antagonists (VKAs), these life-threatening bleeds have been observed in DOAC-treated patients in randomized trials at a rate of approximately 0.7%/year [5]. The literature shows that intracerebral hemorrhage associated with antithrombotic use, when it occurs, can result in a high burden of adverse clinical outcomes, with a hospital mortality of 12.4% for traumatic intracranial hemorrhage and 29% for nontraumatic intracranial hemorrhage [6].

The American Heart Association and Stroke Guidelines also found a high hospital mortality of 27% for patients with spontaneous intracerebral hemorrhage in the presence of FXa inhibitors, with mortality ratios significantly higher in patients taking FXa inhibitors than in patients not taking anticoagulants, but significantly lower than in patients taking warfarin, suggesting that anticoagulants with different mechanisms of action may have different prognoses for the same outcome [7].

In 2018 and 2019, respectively, the first specific FXa inhibitor reversal agent, recombinant FXa (Andexanet alfa), was approved by the FDA and conditionally approved by the EMA for patients treated with rivaroxaban or apixaban when anticoagulation reversal is required for patients with uncontrolled or life-threatening bleeding [8].

Andexanet alfa is a modified recombinant inactive form of human FXa designed as a “decoy” to bind FXa-inhibiting molecules and reduce anti-FXa activity [9]. In the prospective single-arm study evaluating the efficacy of Andexanet alfa (ANNEXA-4), treatment with the drug resulted in a 92% reduction in anti-FXa activity and hemostatic efficacy was observed in 82 of patients [8].

On the other hand, coagulation factor concentrates, mainly four-factor pro-thrombin complex concentrate (4F-PCC), have been used to treat bleeding associated with vitamin K antagonists, replacing vitamin K-dependent coagulation factors (II, VII, IX and X) [10]. In some countries, it has also been used as an alternative strategy for the treatment of severe bleeding associated with FXa inhibitors, although there is a lack of prospective data or clinical trials [11,12].

Currently, there is limited evidence on the comparative efficacy and safety of Andexanet alfa and 4F-PCC in the treatment of intracranial hemorrhage (ICH), both intraoperatively in patients undergoing emergency neurosurgical treatment and in an intensive care unit. Therefore, this systematic review aims to evaluate this data gap through retrospective studies. Therefore, we conducted an indirect comparative study comparing the efficacy and safety of Andexanet alfa and 4F-PCC in the treatment of intracranial hemorrhage associated with FXa inhibitors.

## 2. Materials and Methods

This work was conducted according to the Cochrane Handbook and in compliance with the Preferred Reporting Items for Systematic Reviews and Meta-Analyses (PRISMA) guidelines (Supplementary Table S1) [13]. The protocol was registered at the International Prospective Register of Systematic Reviews (PROSPERO; registration No. CRD42024497914) on 9 December 2023.

### 2.1. Eligibility Criteria

The criteria selection was established following the PICO model (population: patients using DOACs; intervention and control: patients treated with AA or 4F-PCC; and outcome: hemorrhage or thrombosis).

Inclusion of the studies in this systematic review was restricted to papers that met all the following eligibility criteria: (1) retrospective studies; (2) comparing AA to 4F-PCC; (3) patients who used FXa inhibitors; (4) patients with age over or equal to 18 years old; and (5) paper in English and published after 2018. In addition, studies were included only if they reported any of the clinical outcomes of interest. We excluded studies with (1) evaluation by only one group (AA or 4F-PCC); (2) pediatric patients; (3) case reports, reviews, qualitative studies, or protocols; or (4) if they were not published in English.

### 2.2. Search Strategy and Data Extraction

We systematically searched PubMed, Embase, Web of Science (Clarivate Analytics) and the Cochrane Central Register of Controlled Trials up to October 2023 with the following search terms: (“intracranial hemorrhage” OR “ICH”) AND (“rivaroxaban” OR “apixaban”) AND (“andexanet alfa”) AND (“4-factor prothrombin complex concentrate” OR “4F-PCC” OR “4FPCC”).

All studies identified via the search strategy were uploaded into Zotero. Two blinded reviewers (R.A.L.O. and J.M.L.F.) independently screened the studies for titles and abstracts using Zotero (zotero.org). Studies that could not be included based on title and abstract were moved for full-text review. Full-text screening, data extraction and quality assessment were performed by 2 reviewers (R.A.L.O. and J.M.L.F.), and a third reviewer (R.A.C.) resolved any discrepancies. Studies during the full-text review that did not meet all inclusion criteria were excluded.

A standardized table was used for data extraction of the included studies. Extracted information included study identification (author and year), the total number of participants, groups, mean age of participants, trauma (yes or not), concomitant use of antiplatelet drugs and other anticoagulants, mortality and thrombotic events.

Primary and secondary outcomes were also extracted via standardized forms. The primary outcome was hemostatic efficacy determined by stop in the growth of intracerebral hematoma volume. The secondary outcome was an evaluation of mortality and thrombotic events. Thrombotic events were defined in alignment with the reporting paper.

### 2.3. Data Extraction and Quality Assessment

Data extraction of included full-text articles was divided among four reviewers (R.A.L.O., J.M.L.F., G.E.C. and V.W.V.) which were synthesized into one summary table. Two independent reviewers (V.W.V. and L.O.F.) assessed the risk of bias for included studies. The Newcastle–Ottawa (NOS) scale was used for observational studies (Supplementary Table S2).

## 3. Results

### 3.1. Baseline Characteristics

**Figure 1:**
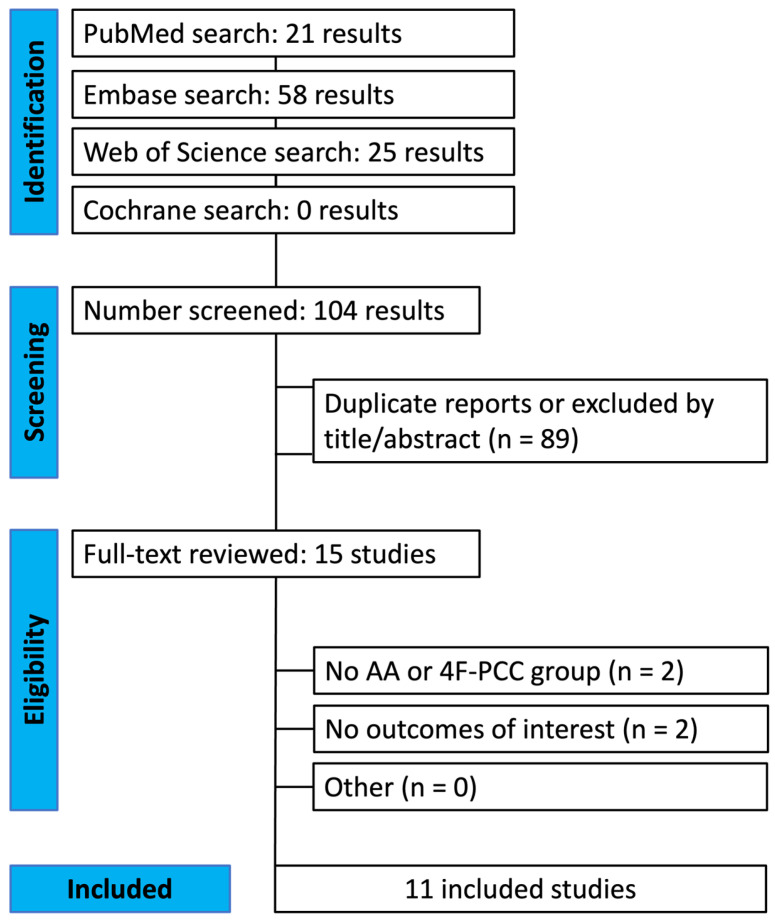
PRISMA flow diagram of study screening and selection.

Our systematic search yielded 104 potential articles, as shown in Figure 1. After removing duplicate records and studies with an exclusion criterion based on title/abstract review, we were left with 15 that were thoroughly screened for inclusion and exclusion criteria. Ultimately, 11 studies were included.

The total number of patients examined in the studies was 1996, with approximately 50% of the patients being female. It is interesting to note that, with the exception of the study by Stevens et al. [14], the average age of patients was always over 70 years, indicating a distinct geriatric population profile and, therefore, more prone to falls/trauma, in greater numbers and to a greater extent than in the general population.

Another important point is the dose set for treatment. In the initial studies with Andexanet alfa, two treatment regimens were established: low dose (LD; 400 mg bolus + 440 mg infusion) and high dose (HD; 800 mg bolus + 860 mg infusion). These regimens take into account two factors: the dose of the factor Xa inhibitor and the time at which this dose was administered at the time of the event [8]. In addition, the concomitant use of other drugs that reduce platelet aggregation may worsen the prognosis of patients compared to those who do not take them. This information is listed in Table 1.

### 3.2. Hemostatic Efficacy

Although several parameters can be used to assess bleeding control, clot stabilization on neuroimaging is the most recommended in patients with ICH. From this point of view, the study by Ammar et al. [15] found no significant differences in the assessment of bleeding stabilization by neuroimaging between patients with ICH receiving AA and 4F-PCC at 6 and 24 h. Although the author performed a subgroup analysis including age and gender, the result remains the same. Lipsky et al. [19] follow the same line of results; while the data presented are promising and show rates of good and excellent bleeding control with the use of AA and 4F-PCC (75% and 66.7%, respectively) within 12 h of drug use, no significant superiority was found with respect to either drug.

In addition, the data from Stevens et al. [14] have important similarities with previous studies, as they did not show superiority of any of the treatments. The results showed that although hemostatic efficacy occurred within 12 h of administration and was achieved in 12 patients (75%) in the Andexanet alfa group compared to 10 patients (62.5%) in the 4F-PCC group, superiority of treatment was not demonstrated. In both groups, hemostasis was rated as excellent in 62.5%, although in the Andexanet alfa group, 12.5% had good hemostasis and 25% had poor hemostasis.

The results of Parsels et al. [21], Vestal et al. [24] and Pham et al. [22] also showed no difference in cerebral hemorrhage volume after 12 and 24 h of treatment (AA vs. 4F-PCC). Oh et al. [20] also came to similar conclusions as previous studies. Although these results are not encouraging, it must be kept in mind that the number of patients examined in the studies was small, implying a sample bias.

On the other hand, the study by Costa et al. [17] showed superiority in the use of Andexanet alfa with a 2.73-fold higher proportion of hemostatic controls in neuroimaging compared to 4F-PCC. This is the result of excellent/good hemostatic efficacy in the overall cohort analysis. Two patients with 4F-PCC with minimal change in hematoma size at follow-up were categorized as having poor/no hemostatic efficacy due to the development of new intraventricular and/or intracerebral hemorrhage after repeat CT scan.

In addition, data presented by Singer et al. [23] showed that hemostatic efficacy was rated as excellent in 80% of patients treated with Andexanet alfa and in 60% of patients treated with 4F-PCC. This result also reflects the survival rate to hospital discharge in patients treated with Andexanet alfa and 4F-PCC, which were 92% and 76%, respectively. Although both results were not significant, the data are promising and encouraging as the population used in this study was homogeneous in terms of baseline characteristics.

From the same perspective, data from Dobesh et al. [18] showed that hematoma volume was smaller in patients taking Andexanet alfa than in patients in the 4F-PCC cohort (22.6 mL and 27.5 mL, respectively). Although no multivariate analysis was performed, these data exhibit selection bias as they were analyzed among different populations with different comorbidities, isolated or combined.

### 3.3. Mortality and Thrombotic Events

Mortality is an important parameter to evaluate the change in outcomes when using a particular drug. In this review, we treat mortality as a secondary outcome. In this sense, the results of Ammar et al. [15] showed that mortality was the same for both treatment groups at 38% and 36% for 4F-PCC and AA, respectively. Pham et al. [22] and Lipski et al. [19] also presented similar results. While Pham et al. [22] observed an overall mortality of 34% and 21% for AA and 4F-PCC, respectively, Lipski et al. [19] presented mortality data up to a follow-up period of 28 days (AA = 39.1; 4F-PCC: 40.4), although this period did not reveal any statistical differences in mortality.

Although the above studies found no difference in mortality, the results of Dobesh et al. [18] showed the opposite. In patients with ICH, in-hospital mortality occurred in 12.6% of patients in the Andexanet alfa group compared to 23.3% of patients in the 4F-PCC group, with an odds ratio of 0.55 (95% CI, 0.39–0.76), i.e., the probability of dying during hospitalization were 45% lower with Andexanet alfa compared to 4F-PCC. Other studies also point in the same direction, showing the superiority of AA over 4F-PCC in terms of mortality, such as the studies by Barra et al. [16] and Costa et al. [17], in which the number of deaths was reduced 3-fold and 2.5-fold, respectively, in patients receiving Andexanet alfa. It is important to emphasize that these deaths were due to the exacerbation of intracranial hemorrhage.

Another important piece of information: although there was no significant difference, all patients in the AA group survived, while those treated with 4F-PCC had a 30-day mortality rate of 13.3% [20]. Stevens et al. [14] and Vestal et al. [24] also present data on 30-day mortality, with higher mortality in the 4F-PCC group than in the Andexanet alfa-treated group (31.5% and 12.5%, respectively), although the AA group had a lower neurological assessment as measured by EEG.

In terms of thromboembolic events, Ammar et al. [15] and Costa et al. [17] did not report thrombotic events associated with 4F-PCC, although two events occurred in the AA group in both studies, which were deep vein thrombosis (DVT). Barra et al. [16], Parsels et al. [21], Singer et al. [23] and Stevens et al. [14] also showed a greater number of thrombotic events with the use of AA compared with 4F-PCC (16.7%, 26.9%, 12% and 25%, respectively).

In contrast, in the data of Vestal et al. [24], Pham et al. [22] and Oh et al. [20], the number of thrombotic events was higher in the group receiving 4F-PCC, with more than 80% of them related to cerebral ischemia and DVT. Lipski et al. [19] also reported a greater number of thromboembolic events with the use of 4F-PCC (8 events) than with AA (5 events), which were related to deep vein thrombosis/pulmonary embolism. It is important to emphasize that the number of adverse events may be related to the size of the population and the safety profile of the drug. Lipski et al. [19] also showed a sample bias in the analysis of adverse events, because while the 4F-PCC group included 47 subjects, the AA group included only 23.

## 4. Discussion

The present study included the qualitative analysis of 11 studies with a total of 1996 patients who had intracranial hemorrhage associated with FXa inhibitor use. We show that although some studies show no superiority or non-inferiority of one drug compared to another, other studies show a 2.7-fold greater likelihood of achieving hemostatic efficacy and a 64% reduction in the relative likelihood of all-cause mortality within 30 days with Andexanet alfa compared to 4F-PCC [17].

The incidence of thrombotic events was not significantly different between groups in any of the articles analyzed. However, a sensitivity assessment considering the topography of the event, such as single-chamber hemorrhage, intracerebral and/or intraventricular hemorrhage, age and gender, could yield different results of hemostatic efficacy or risk of adverse events.

Although the change in bleeding volume between patients using Andexanet alfa compared with 4F-PCC was not significant in the results of Dobesh et al. [18], it is possible that the small sample size was insufficient to detect differences between treatment arms, which is a bias that needs to be considered when extrapolating the data to other populations. Barra et al. [16] reported a case series of patients with intracranial hemorrhage associated with the use of apixaban or rivaroxaban treated with Andexanet alfa or 4F-PCC, and that treatment with AA achieved hemostatic efficacy (defined as a ≤35% increase in the thickness of the subarachnoid or subdural hematoma or the volume of intracerebral hemorrhage). These data are important because different studies have different methods for evaluating hemostatic efficacy [14,15,17,19,20,21,22]; if the methods were standardized, we believe the results of the analyses might represent a different outcome.

Mortality data show differences among studies, while some observed no difference between the two treatments (AA or 4F-PCC), others show significant results and with promising statistics. However, because some studies present a smaller number of patients and variations in mortality assessment time, quantitative comparisons among articles may not be possible. Our analyses are based on indirect comparisons, measuring outcomes in different articles/populations with intracranial hemorrhage. Several international medical societies have developed guidelines about how best to treat intracranial hemorrhage associated with FXa inhibitors. Current guidelines from the American Heart Association/American College of Cardiology/Heart Rhythm Society [25], European Society of Cardiology [26], American College of Emergency Physicians [27], American Society of Hematology [28] and European Stroke Organization Brain [29] each recommends the use of Andexanet alfa for the treatment of severe life-threatening bleeding related to apixaban or rivaroxaban, with some [26,29], but not all, recommending the use of 4F-PCC when Andexanet alfa is not available or to prevent life-threatening bleeding. The last update we had on bleeding control was in the guideline of the European Society of Anesthesiology in its update for the management of severe peri-operative bleeding: Guidelines from the European Society of Anaesthesiology and Intensive Care: Second update 2022 [30], reinforcing the idea of the efficiency of Andexanet alfa; however, it also highlights the increased thrombotic risk.

In this sense, a limitation of this review is the fact that not all studies included edoxaban. Although rivaroxaban and apixaban are more commonly used in the literature, there are already a small number of articles observing the behavior of both treatments (AA or 4F-PCC) in patients with ICH using edoxaban, but with evidence that is not yet very robust [31,32]. Another limitation is the type of studies analyzed. As these are retrospective observational studies, the risk of bias is greater, which means that the interpretation of some results must be done with great caution.

An interesting detail is that the studies do not provide justifications for the use of each DOAC, as a careful assessment of patient characteristics and bleeding risk would be important and fundamental for the selection of the most appropriate DOAC to minimize the risk of bleeding complications.

Therefore, further research needs to be conducted to explore the therapeutic potential of both drugs (AA or 4F-PCC) to outline the best pharmacologic strategy for the treatment of intracranial hemorrhage, whether in emergency surgery or in an intensive care unit.

## Figures and Tables

**Table 1:** Summary of article information. AA: Andexanet alfa. 4F-PCC: four-factor prothrombin complex concentrate. APL: according to the product labeling. LD: low dose. HD: high dose. AC/AP: anticoagulants and antiplatelet.

Study	Year	Groups (*n*)	Average Age (Years)	Trauma (*n*)	Dose (*n*)	Use AC/AP (*n*)	Mortality (*n*)	TE Events (*n*)
Ammar et al. [15]	2021	AA = 28 4F-PCC = 16	AA = 78 4F-PCC = 80	AA = 8 4F-PCC = 8	AA = APL 4F-PCC = 25 U/kg	AA = 13 4F-PCC = 1	AA = 11 4F-PCC = 6	AA = 2 4F-PCC = 0
Barra et al. [16]	2020	AA = 18 4F-PCC = 11	AA = 83.4 4F-PCC = 71	AA = 12 4F-PCC = 5	AA = LD 4F-PCC = 2500 U	AA = 5 4F-PCC = 4	AA = 4 4F-PCC = 7	AA = 3 4F-PCC = 1
Costa et al. [17]	2022	AA = 107 4F-PCC = 95	AA = 79 4F-PCC = 77	AA = 57 4F-PCC = 70	AA = LD (104)/HD (3) 4F-PCC = 25 U/kg (71)/50 U/kg (24)	AA = 36 4F-PCC = 23	AA = 10 4F-PCC = 14	AA = 2 4F-PCC = 0
Dobesh et al. [18]	2023	AA = 666 4F-PCC = 662	AA = 65.6 4F-PCC = 66.6	AA = 327 4F-PCC = 366	No record found	No record found	AA = 84 4F-PCC = 151	No record found
Lipski et al. [19]	2022	AA = 23 4F-PCC = 47	AA = 80 4F-PCC = 81	AA = 16 4F-PCC = 32	AA = LD (17)/HD (6) 4F-PCC = 50 U/kg	AA = 11 4F-PCC = 24	AA = 9 4F-PCC = 19	AA = 5 4F-PCC = 8
Oh et al. [20]	2023	AA = 9 4F-PCC = 15	AA = 82 4F-PCC = 85	No record found	AA = LD (7)/HD (2) 4F-PCC = 50 U/kg	AA = 3 4F-PCC = 6	AA = 0 4F-PCC = 2	AA = 0 4F-PCC = 2
Parsels et al. [21]	2023	AA = 26 4F-PCC = 26	AA = 83 4F-PCC = 77	AA = 16 4F-PCC = 18	AA = LD 4F-PCC = 50 U/kg	AA = 7 4F-PCC = 6	No record found	AA = 7 4F-PCC = 3
Pham et al. [22]	2022	AA = 47 4F-PCC = 62	AA = 77 4F-PCC = 81	No record found	AA = APL 4F-PCC = 50 U/kg	AA = 14 4F-PCC = 22	AA = 16 4F-PCC = 13	AA = 4 4F-PCC = 6
Singer et al. [23]	2023	AA = 25 4F-PCC = 25	AA = 77 4F-PCC = 73	No record found	AA = APL 4F-PCC C = Fab.	No record found	AA = 2 4F-PCC = 6	AA = 3 4F-PCC = 2
Stevens et al. [14]	2021	AA = 16 4F-PCC = 16	AA = 69.1 4F-PCC = 69	No record found	AA = LD (14)/HD (2) 4F-PCC = APL	AA = 5 4F-PCC = 3	AA = 2 4F-PCC = 5	AA = 4 4F-PCC = 3
Vestal et al. [24]	2022	AA = 21 4F-PCC = 35	AA = 73.29 4F-PCC = 74.51	AA = 5 4F-PCC = 11	AA = LD (20)/HD (1) 4F-PCC = 50 U/kg	No record found	AA = 6 4F-PCC = 14	AA = 3 4F-PCC = 11

## Data Availability

Please contact the corresponding author for any inquiries regarding data access.

## Supplementary Materials

The following supporting information can be downloaded at https://www.mdpi.com/article/10.3390/jcm13113077/s1, Table S1: PRISMA 2020 Checklist A Systematic Review; Table S2: Newcastle-Ottawa Quality Assessment Scale Retrospective Studies.
