# Supplementary material for: Andexanet Alfa versus Four-Factor Prothrombin Complex Concentrate for the Reversal of Factor Xa (FXa) Inhibitor-Associated Intracranial Hemorrhage: A Systematic Review of Retrospective Studies
Source: J Clin Med. 2024 May 24;13(11):3077. doi: 10.3390/jcm13113077 (PMC11173120; doi:10.3390/jcm13113077)
Supplement: Supplementary file 1 [file jcm-13-03077-s001.zip › Table S2.pdf]

Supplementary Table S2. Newcastle - Ottawa Quality Assessment Scale Retrospective Studies.

Author, Year	Selection				Comparability	Outcome		
	Representativeness of the treatment (AA)	Representativeness of the treatment (4F-PCC)	Ascertainment of exposure	Is it clear that the binary outcome of interest was not present at start of study	Comparability of cohorts on the basis of the design or analysis	Ascertainment of outcome:	Follow-up long enough for outcomes to occur	Adequacy of follow-up
Ammar et al. 2021	1 star	1 star	1 star	1 star	1 star	1 star	0 stars	1 star
Barra et al. 2020	1 star	0 stars	1 star	1 star	1 star	1 star	1 star	1 star
Costa et al. 2022	1 star	0 stars	1 star	1 star	1 star	1 star	1 star	1 star
Dobesh et al. 2023	1 star	1 star	1 star	1 star	1 star	1 star	1 star	1 star
Lipski et al. 2022	1 star	1 star	1 star	1 star	1 star	1 star	1 star	1 star
Oh et al. 2023	1 star	1 star	1 star	1 star	1 star	1 star	1 star	1 star

Parsels et al. 2023	1 star	1 star	1 star	1 star	1 star	1 star	0 stars	1 star
Pham et al. 2022	1 star	1 star	1 star	1 star	1 star	1 star	0 stars	1 star
Singer et al. 2023	1 star	1 star	1 star	1 star	1 star	1 star	1 star	1 star
Stevens et al. 2021	1 star	1 star	1 star	1 star	1 star	1 star	1 star	1 star
Vestal et al. 2022	1 star	1 star	1 star	1 star	1 star	1 star	1 star	1 star
